# The Autophagy Machinery: A New Player in Chemotactic Cell Migration

**DOI:** 10.3389/fnins.2017.00078

**Published:** 2017-02-16

**Authors:** Pierre-Michaël Coly, Pierrick Gandolfo, Hélène Castel, Fabrice Morin

**Affiliations:** ^1^Normandie Univ, UNIROUEN, Institut National de la Santé et de la Recherche Médicale (INSERM), DC2NRouen, France; ^2^Institute for Research and Innovation in BiomedicineRouen, France

**Keywords:** autophagosome biogenesis, cell adhesion, chemotactic migration, CXCR4, GPCR, urotensin II

## Abstract

Autophagy is a highly conserved self-degradative process that plays a key role in diverse cellular processes such as stress response or differentiation. A growing body of work highlights the direct involvement of autophagy in cell migration and cancer metastasis. Specifically, autophagy has been shown to be involved in modulating cell adhesion dynamics as well as epithelial-to-mesenchymal transition. After providing a general overview of the mechanisms controlling autophagosome biogenesis and cell migration, we discuss how chemotactic G protein-coupled receptors, through the repression of autophagy, may orchestrate membrane trafficking and compartmentation of specific proteins at the cell front in order to support the critical steps of directional migration.

## Chemotactic migration: control by G protein-coupled receptors

### Steps of chemotactic migration

Chemotactic cell migration is a highly coordinated process that is crucial to the function of many cell types. As such, it is a fundamental property of a variety of physiological and pathological phenomena. Chemotactic migration is first observable during embryonic development, as sheets of cells undergo migration to form the different layers of the embryo and later, the different tissues that constitute organs (Keller, 2005). In the central nervous system, chemotactic migration allows cells such as new neurons to localize to the appropriate cortical layer, and guides the elongation of their growth cones to facilitate circuit formation (Cooper, 2013). Cell migration is also involved in immune response and angiogenesis allowing cells to infiltrate and navigate through tissues (Imhof and Dunon, 1997). A few pathological processes can also take advantage of a cell's migration abilities to spread through the organism. This is the case in cancer progression, during which parenchyma invasion and metastasis formation heavily rely on chemotactic migration (Bravo-Cordero et al., 2012).

Previous studies have shown that chemotactic migration can be broken down into a few successive steps. Surface receptors pick up on chemotactic cues in the extracellular environment and orient chemotaxis. These receptors can activate signaling cascades that establish a front-rear polarity. The class I phosphatidylinositol 3-kinase (PI3K) is a vital player during this step. The lipid kinase forms phosphatidylinositol (3,4,5)-triphosphate (PIP3) at the cell front, which serves as a signal for several pathways that converge toward reorganizing the actin cytoskeleton (Weiner, 2002). This allows the formation of actin-dependent membrane protrusions toward the chemotactic signal. The lamellipodium is probably the most characterized type of cell protrusion involved in migration. It is composed of a dense dendritic network of actin filaments that pushes the plasma membrane forward, but also serves as an intracellular scaffold favoring the appearance of links with the extracellular matrix (ECM). These links, called adhesion complexes, stabilize the lamellipodium and act as a molecular clutch allowing the cell body to pull itself forward (Ridley et al., 2003).

Adhesion complexes are highly dynamic structures that are formed by the hierarchical recruitment of different scaffolding proteins. At their base we find integrins, heterodimer transmembrane proteins with long extracellular heads that can bind to ECM components such as fibronectin. This link, coupled with intracellular cues, activates integrins by modifying the conformation of their extracellular heads, thereby increasing their affinity for the ECM (Tadokoro et al., 2003; Campbell and Humphries, 2011). Activated integrins also cluster together to form more robust structures that are linked to actin filaments by talin and paxillin (Ridley et al., 2003). During migration, the retrograde flow of actin filaments applies forces to the adhesion complexes that seem to be essential to their sustainability and maturation, by allowing the addition of strengthening proteins such as vinculin (Choi et al., 2008). A rearward motion of these adhesion complexes can be observed as the cell body moves forward. Once they reach a certain point, they begin to disassemble, as to not impede with migration. Cells that cannot effectively disassemble adhesion complexes are considerably slowed in their advance since they cannot detach from the substratum (Kaverina et al., 1999; Ezratty et al., 2005). Adhesion turnover also serves to recycle adhesion proteins to the cell front so that they may aid in the construction of new complexes (Margadant et al., 2011).

Disassembly seems to rely on “relaxation” signals carried by microtubules. Early observations showed that adhesions are destabilized as microtubules grow toward them. These filaments may in fact stimulate loss of tension by bringing focal adhesion kinase (FAK) and calpains to the adhesion site. Phosphorylation of paxillin by FAK contributes to the weakening of the structure, whereas calpains physically disrupt the link between actin and the ECM by cleaving talin (Franco S. J. et al., 2004; Webb et al., 2004). Ubiquitination also seems to partake in adhesion disassembly, as several proteins, such as FAK, paxillin, and integrins are ubiquitinated during this process (Huang, 2014). The final step involves the endocytosis of integrins, mainly by a clathrin-dependent pathway (Ezratty et al., 2009). Once internalized, integrins can either be transported to the cell front for the formation of new adhesion complexes, or directed to autophagosomes and lysosomes for degradation (Tuloup-Minguez et al., 2013; Maritzen et al., 2015).

### Chemotactic G protein-coupled receptors

With over 800 genes in Human, G-protein coupled receptors (GPCR) constitute the largest surface receptor family (Fredriksson et al., 2003). Their role is to help the cell adapt to its environment by translating extracellular cues to intracellular responses. GPCRs are involved in a wide assortment of physiological processes and as such, their ligands vary from hormones to lipids and even photons. Many GPCRs can drive cell migration by enhancing motility and guiding the orientation of actin polymerization and adhesion complexes formation. Three main types of GPCRs have been found to induce chemotaxis. These include receptors for chemokines, some vasoactive peptides and bioactive lipids (Cotton and Claing, 2009).

Chemokines constitute a large family of chemotactic cytokines that can stimulate directed cell migration upon binding to their GPCR. They are characterized by the presence of four cysteine residues in their sequence and are named according to the position of the two first ones. Therefore, they are classified in four groups (CC, CXC, C, and CX3C) which bind to GPCRs named accordingly (Murphy et al., 2000). Few chemokine GPCRs have received as much attention as the C-X-C motif chemokine receptor 4 (CXCR4). CXCR4 plays pleiotropic functions in the peripheral immune system by stimulating the migration of monocytes and lymphocytes (Bleul et al., 1996). It is also an important regulator for homing of hematopoietic progenitor cells to the bone marrow microenvironment (Lapidot et al., 2005). In the central nervous system, CXCR4 participates in guiding developing interneurons to their proper cortical layer, as well as recruiting microglial cells during cortical development (Li and Ransohoff, 2008; Tiveron and Cremer, 2008; Nash and Meucci, 2014). Moreover, in CXCR4^−/−^ mice, most GnRH neurons fail to exit the vomeronasal organ during embryonic development, and comparatively few GnRH neurons reach the forebrain (Schwarting et al., 2006). In the adult brain, this GPCR is believed to influence regeneration by recruiting brain-resident and circulating cells to the site of the lesion (Stumm and Höllt, 2007). CXCR4 is also notable for its involvement in the internalization of the HIV as well as in the progression of a wide range of cancers (Feng et al., 1996; Chatterjee et al., 2014). As such, studies have shown that CXCR4 increases the migration rate of several types of cancer cells (Salcedo et al., 2003).

A few vasoactive peptides, initially characterized for the effects on the cardiovascular system, have more recently been shown to increase cell migration. For example, by binding to their cognate GPCRs, angiotensin II and endothelins can drive the migration of smooth muscle cells and endothelial cells (Xi et al., 1999; Daher et al., 2008). Urotensin II, the most potent vasoactive peptide identified so far, is able to induce directed cell migration of monocytes, endothelial cells as well as glioma cells (Segain et al., 2007; Xu et al., 2009; Brulé et al., 2014; Lecointre et al., 2015). Several bioactive lipids have also been found to induce chemotaxis. One of them is lysophosphatidic acid, which, in the nervous system, has been shown to stimulate the migration of embryonic schwann cells and astrocytes (Sato et al., 2011; Anliker et al., 2013; Yung et al., 2015). Lysophosphatidic acid also drastically accelerates tumor growth by inducing angiogenesis and tumor invasion, two processes that rely on increased migration (Contos et al., 2000; Blackburn and Mansell, 2012).

Chemotactic GPCRs initiate signaling cascades that regulate cell migration by activating heterotrimeric G proteins, composed of three subunits, α, β, and γ. Activation of a GPCR switches out the GDP for a GTP in the Gα subunit, which causes the dissociation of Gα from Gβγ. Each subunit can then go on to regulate different intracellular signaling pathways (Wilkie et al., 1992). Based on their sequences, Gα proteins can be split into four main subtypes: α_s_, α_i/o_, α_q/11_, and α_12/13_ (Simon et al., 1991). Though previous studies have shown that all of these subtypes can, in one way or another, modulate cell migration, it appears that GPCR-induced chemotaxis is mainly relayed by Gα_i_ and Gα_12/13_ (Cotton and Claing, 2009; Lecointre et al., 2015). These G proteins have been linked to the activation of GTPases belonging to the Rho family: RhoA, Rac1, and Cdc42. Together, the GTPases orchestrate the construction of the dendritic actin network in the lamellipodium, as well as the formation and maturation of adhesions.

## The autophagy machinery

### General mechanisms of autophagosome biogenesis

Macroautophagy (hereafter referred to as autophagy) is an evolutionarily conserved lysosomal pathway involved in the degradation of long lived proteins and cytoplasmic organelles (Hale et al., 2013). This process, which is essential for normal turnover of cellular compartments, is up-regulated in response to nutrient starvation. The mechanistic target of rapamycin (mTOR) kinase is a key regulator of cell metabolism that represses autophagic activity when nutrient conditions are adequate. mTOR is itself inhibited upon nutrient starvation, which results in autophagy induction (Kim et al., 2011). One of the first events in autophagy is the formation of the phagophore, a cup-shaped isolation membrane. The edges of these phagophore membranes elongate and thereby engulf portions of cytoplasm. After the fusion of the membrane edges, the structure becomes a completed autophagosome, which later fuses with lysosomes, resulting in the degradation of its luminal content. Several highly conserved autophagy (ATG) proteins, which control key steps in the autophagy process, have been identified (Nakatogawa et al., 2009). Initiation of the phagophore requires the Beclin1-containing class-III PI3K complex, generation of phosphatidylinositol 3-phosphate (PI3P), and recruitment of the PI3P-binding proteins called WD repeat domain phosphoinositide interacting (WIPI) and double FYVE domain- containing protein 1 (DFCP1). These are followed by the recruitment of the ATG5-ATG12-ATG16L1 ternary complex, along with phosphatidylethanolamine-conjugated microtubule-associated protein 1 light chain 3 beta (LC3-II), which are essential for elongation of the phagophore membrane. While the ATG5-ATG12-ATG16L1 complex decorates the phagophore and dissociates after completion of autophagosome formation, part of LC3-II remains associated with fully formed autophagosomes (Abada and Elazar, 2014). In addition to its bulk degradation property, autophagy also partakes in the clearance of specific substrates. This selective autophagy mainly depends on cargo receptors such as neighbor of BRCA1 gene 1 (NBR1) and p62, which can bind to ubiquitin-tagged substrates. These cargo receptors can also bind to LC3 *via* a LC3-interacting region (LIR) motif, which therefore targets them to autophagosomes (Bjørkøy et al., 2005; Pankiv et al., 2007; Kirkin et al., 2009; Zaffagnini and Martens, 2016).

### Sources of membrane for the expansion of the phagophore

Phagophores require lipids to mature into autophagosomes. After more than 50 years of investigations, the origin of the autophagosomal membranes is still a critical question. Originally, the endoplasmic reticulum (ER) was proposed to be the primary source of these membranes. Early electron microscopy studies identified a close relationship between the ER and autophagic structures, suggesting that autophagosomal membranes are mainly delivered from the ER (Novikoff and Shin, 1978; Hayashi-Nishino et al., 2009). Consistent with this idea, Axe et al. (2008) showed that, in response to amino acid starvation, the PI3P-binding protein DFCP1, translocates to PI3P-enriched subdomains of the ER. These subdomains then constitute a platform for accumulation of autophagosomal proteins, expansion of autophagosomal membranes and emergence of fully formed autophagosomes. Subsequent three-dimensional tomography studies (Hayashi-Nishino et al., 2009; Ylä-Anttila et al., 2009) demonstrated that subdomains of the ER form a cradle-like curve encircling isolation membranes. The associated ER and isolation membranes are interconnected by a narrow membrane extension from the isolation membrane. Recent studies found evidence that apart from the ER, numerous other membrane sources are involved in the formation of autophagosomes, including mitochondria, the Golgi, recycling endosomes and endocytic vesicles budding from the plasma membrane. Hailey et al. (2010) elegantly demonstrated that, in starved cells, mitochondria directly participate in autophagosome biogenesis. They found that the early autophagosomal maker, ATG5, transiently localizes to puncta on mitochondria, followed by the late autophagosomal marker LC3. This study further showed that cell starvation drives the delivery of lipid components from the mitochondrial outer membrane to newly formed autophagosomes. It has recently been reported that the Golgi may also contribute to the formation of autophagosomes. Following starvation, activation of the class-III PI3K complex promotes re-localization of COPII adaptors from the ER exit sites to the ER-Golgi intermediate compartment (ERGIC). The process leads to the generation of ERGIC-derived COPII vesicles which becomes LC3-positive and contribute to autophagosome biogenesis (Ge et al., 2015).

Recent reports demonstrated that recycling endosomes, through the formation of tubular structures accumulating autophagy proteins, also supply membrane for autophagosome biogenesis. In a siRNA-mediated screen, Knaevelsrud et al. identified the PX domain-containing protein, SNX18, as a positive regulator of autophagy (Knævelsrud et al., 2013). The membrane binding and tubulation activities of SNX18, as well as its direct interaction with LC3, allow the formation of LC3-ATG16L1-positive tubules emanating for recycling endosomes that provide membrane input to forming autophagosomes. This study is in line with other findings (Longatti et al., 2012) showing that vesicular transport from recycling endosomes, negatively regulated by the Rab11 effector protein TBC1D14, contributes to starvation-induced autophagy. Together, these data indicate that the recycling compartment is not solely responsible for recycling of plasma membrane receptors but also serves as a sorting station for controlled delivery of membrane for autophagosome biogenesis.

Work from Rubinsztein's lab identified endocytic vesicles, trafficking to recycling endosomes, as an important source of membrane for autophagosome biogenesis. Endocytic vesicles can form from regions of the plasma membrane through different mechanisms, i.e., clathrin-dependent and clathrin-independent vesicle budding (Ravikumar et al., 2010; Moreau et al., 2012). Accumulation of ATG16L1 at clathrin-coated endocytic structures, through an interaction between ATG16L1 and the clathrin adaptor AP2, and vesiculation of ATG16L1-positive precursors have been found to contribute to autophagosome formation. Inhibition of clathrin-mediated endocytosis, using siRNAs targeting the clathrin heavy-chain or the clathrin adaptor AP2, causes defective autophagosome biogenesis, which is associated with impaired uptake of plasma membrane into pre-autophagosomal vesicles (Ravikumar et al., 2010). These ATG16L1-positive vesicles then undergo SNARE-mediated homotypic fusion, generating tubulovesicular structures that increase in size, enabling the acquisition of LC3 protein (Moreau et al., 2011) Similarly to ATG16L1-positive vesicles, generation of clathrin-coated ATG9-positive vesicles from the plasma membrane also participates in autophagosome formation. Surprisingly, ATG16L1 and ATG9 proteins have been found to localize to distinct clathrin-coated vesicles and to traffic through different routes inside the cell. Although both ATG9 and ATG16L1 proteins end up in recycling endosomes, ATG9 is trafficked via EEA1-positive early endosomes, whereas ATG16L1 has minimal residence in early endosomes (Puri et al., 2013; Zavodszky et al., 2013). The SNARE protein named VAMP3, which co-traffics with ATG9, seems to be critical for the coalescence of ATG16L1 and ATG9 vesicles in recycling endosomes (Puri et al., 2013). The impact of this coalescence on the formation of tubules emanating from recycling endosomes, driven by SNX18, deserves further investigations.

## Regulation of the autophagy machinery by G protein-coupled receptors

To this day, very few GPCRs have been shown to directly affect autophagic activity. These mainly include nutrient sensing receptors that increase anabolic processes via stimulation of the mTOR kinase, a well-known autophagy repressor (Jung et al., 2010; Wauson et al., 2014). The amino-acid responsive T1R1/T1R3 receptor is present in most tissues and acts as a sensor for the fed state and amino acid availability. It has been suggested that this GPCR may impact autophagic activity through mTOR stimulation. Reducing T1R3 levels in HeLa cells is sufficient to impair mTOR activity and activate autophagy (Wauson et al., 2012). Angiotensin receptors have also been found to modulate autophagic activity in cardiomyocytes (Porrello et al., 2009), podocytes (Yadav et al., 2010) and in vascular smooth muscle cells (Yu et al., 2014), mainly through the generation of reactive oxygen species.

We recently found that chemotactic GPCRs CXCR4 and the urotensin II receptor (UT) also reduce autophagic activity by inhibiting autophagosome biogenesis (Coly et al., 2016). Unlike the studies cited above, these anti-autophagic effects do not seem to be relayed by mTOR modulation, but rather by inhibiting ATG16L1 recruitment to pre-autophagic vesicles budding from the plasma membrane. While Ravikumar et al. (Bjørkøy et al., 2005) demonstrated that ATG16L1 recruitment is dependent on its interaction with the AP2-clathrin complex, the data we obtained indicate that ATG5 is also implicated. We demonstrated that activation of CXCR4 or UT reduces the pool of ATG5 protein located at the plasma membrane, thereby reducing the recruitment of ATG16L1. Accordingly, overexpression of recombinant ATG5 totally abrogates the anti-autophagic activities of CXCR4 and UT, and siRNA-mediated knockdown of *ATG5* mimics the inhibitory effects of these GPCRs on the formation of pre-autophagic endosomes. What is the exact role of ATG5 in mediating the formation of pre-autophagic endosomes? We can speculate that ATG5's membrane binding activity (Romanov et al., 2012) might allow the initial docking of an ATG5-ATG16L1 complex to the plasma membrane in order to maximize the probability of interaction between ATG16L1 and AP2-clathrin. Alternatively, since ATG5 can co-immunoprecipitate from cell lysates with ATG16L1 and clathrin, and since the N-terminus region of ATG16L1 allows both AP2-clathrin co-immunoprecipitation (Ravikumar et al., 2010) and direct ATG5 binding (Mizushima et al., 1999; Otomo et al., 2013; Kim et al., 2015), it is conceivable that ATG5 may act as a bridge between ATG16L1 and AP2-clathrin.

## G protein-coupled receptor-induced activation of calpains: a critical event that relays pro-migratory and anti-autophagic properties

### Pro-migratory properties of calpains

Calpains are a ubiquitously expressed family of cysteine proteases that mediate cleavage of specific substrates. Although calpain proteolysis can lead to full degradation of some of its substrates, others are cleaved in a limited fashion, resulting in protein fragments that have altered distributions and/or functions. Calpains have thus been found to be involved in a number of processes such as development, cell death, and motility (Goll et al., 2003). Modulating cell migration is one of the better known roles of these proteases. Studies conducted in neutrophils have shown that calpain inhibition increases random migration, but decreases GPCR-induced directional migration upon exposure to a gradient of interleukin 8 (Lokuta et al., 2003). In neurons, calpain activity was also shown to be essential for SDF1-induced actin reorganization and directional migration (Lysko et al., 2014). These results are in line with work highlighting the role of the calpain 2 isoform during lamellipodium formation. Calpain 2 controls the formation of cell protrusions by cleaving cortactin, a key modulator of actin filament branching at the cell front. Expression of a calpain-resistant form of cortactin reduces the migration of fibroblasts by increasing the number of transient and inefficient cell protrusions (Perrin et al., 2006). Calpains also play an important role in the dynamics of adhesion formation and disassembly. By modifying the cytoplasmic tail of β-integrins, calpains seem to be essential for the formation of integrin clusters at an early stage of adhesion complex assembly (Bialkowska et al., 2000) Talin is another calpain target during these initial steps. Once cleaved, talin can bind to β-integrin tails, therefore constituting the first link between integrins and actin filaments (Yan et al., 2001) In addition to their role during this assembly phase, calpains are also one of the main actors of adhesion disassembly. They contribute to adhesion turnover by destabilizing the structural integrity of the complex. Several proteins such as paxillin, vinculin and talin are in fact targeted by calpains during this stage (Carragher et al., 1999; Franco S. et al., 2004; Serrano and Devine, 2004). Inhibiting calpains with either calpastatin or pharmacological means significantly slows adhesion turnover (Bhatt et al., 2002). Similar results can be obtained following calpain 2 knockdown, which results in large, long lasting adhesion complexes that inhibit cell detachment and therefore impair cell migration (Franco S. J. et al., 2004). Despite the many roles of calpains during cell migration, their regulation by chemotactic GPCRs remains unclear. However, previous work revealed that calpain 2 is recruited at the plasma membrane and activated following its phosphorylation by ERK and dephosphorylation on a protein kinase A (PKA) site (Glading et al., 2000; Shiraha et al., 2002). Interestingly, as mentioned earlier, the pro-migratory properties of many chemotactic GPCRs are relayed by G_i_ coupling, which has the ability to activate ERK, through βγ subunits, and to inhibit PKA, through the α_i_ subunit (Goldsmith and Dhanasekaran, 2007; Cotton and Claing, 2009). We can therefore speculate that the simultaneous induction of these signaling pathways by chemotactic GPCRs may be determinant for the activation of calpain 2 at the plasma membrane and regulation of adhesion dynamics.

### Anti-autophagic properties of calpains

A growing amount of data suggests that calpains are major inhibitors of the autophagy machinery. SiRNA-mediated knockdown of calpain 1 is sufficient to induce autophagy under nutrient rich conditions, correlated with increased levels of LC3-II and ATG5-ATG12 complex (Xia et al., 2010). Using a cell-free system, Yousefi et al. demonstrated that ATG5 can be cleaved by both calpain 1 and calpain 2 (Yousefi et al., 2006). Cleavage of ATG5 then generates a 24 kDa N-terminal product that can translocate to the mitochondria and enhance susceptibility toward apoptotic stimuli (Yousefi et al., 2006). *In vitro* experiments also identified ATG3, ATG4, ATG7, ATG9, ATG10, ATG12, and Beclin1 as direct calpain substrates (Norman et al., 2010; Yang et al., 2010). It should be noted that calpains may also exert their anti-autophagic properties by targeting *non-ATG* proteins. The clathrin adaptors AP2 and PICALM, which are critical for the formation of pre-autophagosomal vesicles from the plasma membrane, have been described as calpain substrates (Kim and Kim, 2001; Rudinskiy et al., 2009; Ando et al., 2013). Does calpain-dependent repression of autophagy then constitute a critical event for chemotaxis? In favor of this hypothesis, we found that the anti-autophagic and pro-migratory properties of two chemotactic GPCR, CXCR4, and UT, were abrogated by pharmacological inhibition or siRNA knockdown of calpains (Coly et al., 2016). We further demonstrated that calpain activation, induced by CXCR4 or UT, reduces the pool of ATG5 at the plasma membrane and inhibits the recruitment of ATG16L1 protein to endocytic vesicles, thereby limiting the formation of pre-autophagosomal precursors required for the expansion of the phagophore and formation of mature autophagosomes. In addition to reversing the anti-autophagic effects of chemotactic GPCRs, calpain inhibition or ATG5 overexpression is also sufficient to block their pro-migratory properties, as both these approaches reduce the cells' migration rate, as well as the number of adhesions per cell (Coly et al., 2016). Despite early reports pointing to ATG5 as a calpain target, our attempts at demonstrating its direct cleavage following CXCR4 or UT activation were unsuccessful. One hypothesis is that only a minor, plasma membrane-associated fraction of ATG5 is cleaved by calpains. The cleaved products may also be highly unstable, thereby hindering their detection. Alternatively, the anti-autophagic action of calpains following GPCR activation could depend on the cleavage of the adaptor proteins AP2 and PICALM, or on the cleavage of ATG7, which is essential for conjugation of ATG5–ATG12 (Mizushima et al., 1998). Since the recruitment of calpains at the plasma membrane constitutes an early event during chemotaxis (Franco and Huttenlocher, 2005), it can be anticipated that GPCR-induced inhibition of autophagy may tightly control early steps of cell polarization.

## G protein-coupled receptor-induced inhibition of autophagy: potential impact on chemotactic migration and invasion

### Lamellipodium expansion vs. autophagosome biogenesis: competition for a common source of membrane?

During GPCR-induced chemotactic migration, efficient expansion of the lamellipodium requires addition of extra membrane at the leading edge, through polarized, microtubule-dependent exocytosis (Bretscher and Aguado-Velasco, 1998; Pierini et al., 2000; Schmoranzer et al., 2003). Work from Veale et al. identified VAMP3-positive recycling endosomes as an important source of internal membrane that is incorporated at the leading edge during macrophage migration (Veale et al., 2010). The authors further demonstrated that, in order for this to happen, the R-SNARE VAMP3 needs to form a complex with its cognate Q-SNARE complex Stx4/SNAP23 located at the cell surface. Loss of any one of the components of the VAMP3/Stx4/SNAP23 complex inhibits efficient lamellipodium formation and alters cell migration. Along with the incorporation of extra membrane, this mechanism also allows the recycling of cell adhesion components at the leading edge, including integrins (Veale et al., 2010).

**Figure 1:**
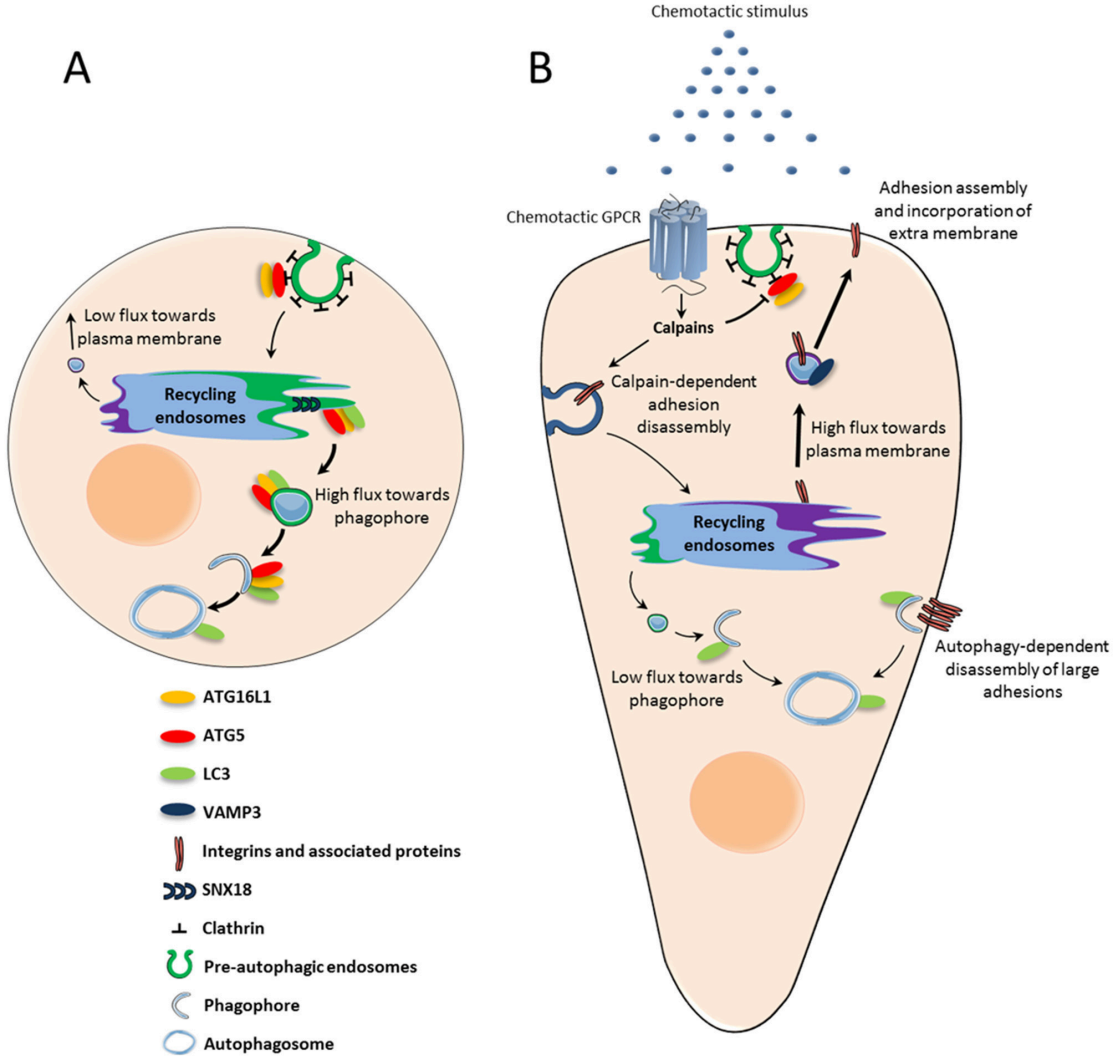
**Chemotactic GPCR-mediated autophagy inhibition: potential role in chemotactic migration. (A)** Under basal conditions, ATG5-ATG16L1-positive pre-autophagic endosomes bud from the plasma membrane and are directed to the recycling endosome compartment. From there, SNX18-dependent tubules target vesicles containing ATG5-ATG16L1 and LC3 to the expanding phagophore. **(B)** Upon activation by chemoattractant stimuli, chemotactic GPCRs locally inhibit the formation of pre-autophagic endosomes. The subsequent reduction of ATG proteins in the recycling compartment may trigger a “targeting switch” which reduces membrane flux toward the phagophore to favor VAMP3-enabled recycling to the plasma membrane. Exocytosis allows integrins to be recycled to nascent adhesions, while phospholipids are incorporated into the lamellipodium and contribute to its expansion. Autophagy inhibition at the leading edge may also locally protect proteins involved in actin remodeling and adhesion assembly, which would otherwise be sequestered and degraded. Autophagy could remain active at distance from chemotactic GPCRs in order to participate in the disassembly of large focal adhesions.

Since recycling endosomes, through the SNX18-dependent formation of tubules, supply membrane for phagophore expansion, it is conceivable that this compartment may constitute a sorting station that deliver phospholipids in a competitive manner, for either lamellipodium expansion or autophagosome synthesis. The dynamic increase in plasma membrane surface triggered by chemotactic GPCRs may then directly impact the pool of phospholipids available for autophagic activity. How could activation of GPCRs, located at the cell surface, affect the trafficking of membrane from recycling endosomes? Chemotactic GPCRs CXCR4 and UT alter, through the activation of calpains, the recruitment of ATG16L1 in pre-autophagosomal vesicles budding from the plasma membrane (Coly et al., 2016). This may reduce the pool of ATG16L1 targeted to the recycling compartment and limit the coalescence of ATG16L1 and ATG9 vesicles. Inhibition of ATG16L1 and ATG9 coalescence would then favor the delivery of VAMP3-positive vesicles at the cell front, at the expense of the phagophore (Figure 1).

### Front cell's accumulation of focal adhesion components

Among the hallmarks of cell migration, the formation of adhesion complexes at the cell's leading edge is among the most notable. Adhesions are critical in generating the traction required for the cell's forward movement. Several data have demonstrated autophagic degradation of key proteins involved in the initiation and the maturation of adhesion complexes, indicating that autophagy can regulate adhesion dynamics. The Src kinase, which is involved in adhesion signaling, was shown to co-immunoprecipitate with LC3 and to be degraded by autophagy (Sandilands et al., 2012). In fibroblasts, β1 integrin-containing vesicles co-localize with LC3-stained autophagic structures. Inhibition of autophagy by ATG5 or ATG3 knockdown is able to slow β1 integrin degradation and to promote it's recycling to the plasma membrane (Tuloup-Minguez et al., 2013). Kenific et al. (Shiraha et al., 2002) recently demonstrated that the selective autophagy cargo receptor NBR1 is essential for adhesion turnover and for the autophagic capture of multiple adhesion proteins including paxillin, vinculin and zyxin. Furthermore, paxillin was shown to have its own LIR domain, which is also involved in its autophagic degradation (Sharifi et al., 2016). In agreement with a role of autophagy in adhesion disassembly, global inhibition of autophagosome biogenesis, using knockdown strategies against ATG proteins, results in the accumulation of large and unproductive adhesions at the entire cell periphery that reduce cell migration (Kenific et al., 2016a,b; Sharifi et al., 2016). Also these migration studies could appear to conflict with our report, (Coly et al., 2016) indicating that autophagy inhibition by CXCR4 or UT stimulates migration, they actually stress the fact that efficient chemotactic migration may imply compartmentalized rather than general inhibition of the autophagic machinery (Lecointre et al., 2015). We can propose that, at the front-most part of the cell, chemotactic GPCRs activated by a gradient of ligand could inhibit autophagy to favor the efficient formation of adhesions, while autophagy would remain active at distance from the site of GPCR activation/signaling in order to enable focal adhesion disassembly.

### Front cell's accumulation of proteins participating in actin remodeling

Chemotactic GPCRs are known to induce actin polymerization at the cell's leading edge to allow the lamellipodium to protrude toward the chemoattractant stimulus. Interestingly, a number of proteins involved in actin dynamics and lamellipodium expansion have been shown to be degraded by autophagy. A Proteomic analysis allowed the identification of the actin regulators twinfilin, WIPF1, cortactin and cofilin 1 in ATG16L1-positive pre-autophagic vesicles budding from the plasma membrane (Morozova et al., 2015). Recent studies also indicate that the Rho GTPases Rac1 and RhoA can be regulated by autophagy. Using keratinocytes, Carroll et al. showed that Rac1 is inactivated during starvation induced autophagy (Carroll et al., 2013). LC3 is able to block Rac1 activation by binding to one of its effectors, Armus. LC3 can also directly interact with Rac1, though whether this leads to Rac1 degradation remains to be determined. Active RhoA and its regulator GEF-H1, can be ubiquitinated and recognized by p62, therefore leading to their selective degradation by the autophagic machinery (Belaid et al., 2014; Yoshida et al., 2016). Autophagy inhibition by shRNA targeting of ATG5 leads to an accumulation of RhoA at the cell surface and to the formation of actin rich lamellipodia. Interestingly, Belaid et al. (Ando et al., 2013) found that the intense actin polymerization caused by RhoA accumulation actually impairs cell motility. Once again, this implies that autophagy inhibition by chemotactic GPCRs may be fine-tuned and compartmentalized at the cell front in order to support effective cell migration.

### Induction of the EMT

Epithelial to mesenchymal transition (EMT) plays a fundamental role in embryonic development and tissue repair. Numerous lines of evidence indicate that EMT also participates in tumor progression and metastasis. Once undergoing EMT, tumoral cells lose their apical-basal polarity, and acquire a mesenchymal phenotype characterized by an elongated morphology and increased motility (Kalluri and Weinberg, 2009). This allows them to detach from the primary site and invade the surrounding tissues and blood vessels. Interestingly, recent publications also link EMT to glioblastoma progression. Although not of epithelial origin, glioblastoma cells can engage an EMT-like process that increases their invasive properties (Kahlert et al., 2013). EMT has been shown to be driven by a variety of signals, such as transforming growth factor-β, insulin growth factor II, or epidermal growth factor (Thiery et al., 2009). These EMT inducers then lead to the activation of core transcription factors, including Snail and Slug, ZEB1/2, and Twist (Tam and Weinberg, 2013).

A complex relationship exists between autophagy and EMT. On one hand, cells that have undergone EMT require increased autophagy to survive stressful environmental conditions during their migration. On the other hand, recent observations indicate that autophagy acts as an oncosuppressive mechanism by inhibiting early steps of EMT (Gugnoni et al., 2016). This latter idea was first proposed by Lv et al. (2012) who demonstrated that, in breast cancer cells, the intracellular signaling protein DEDD (death-effector domain-containing DNA-binding protein) inhibits EMT through the activation of autophagy and consecutive degradation of Snail and Twist. Snail and Twist were found to colocalize with the autophagosomal marker LC3, and inhibition of autophagy using 3-methyladenine significantly reduced their degradation rates (Lv et al., 2012). Using mouse embryonic fibroblast (MEF) cells, Qiang et al. found that ATG3, ATG5, ATG9, or ATG12 knockout cells exhibit much higher invasive properties than wild-type cells (Qiang et al., 2014). The authors demonstrated that autophagy deficiency promotes EMT events through the accumulation of p62 in the cytosol. Accumulating p62 then binds to Twist1 and prevents its proteasomal degradation. A recent study obtained in glioblastoma indicates that autophagy inhibition, through the knockdown of ATG5 or ATG7, stimulates the expression of the EMT regulators Snail and Slug, as well as cell invasion (Catalano et al., 2015).

From these data, it can be expected that inhibition of autophagy by chemotactic GPCRs, such as CXCR4 or UT (Coly et al., 2016), may constitute a critical event participating in EMT during tumor progression. This hypothesis is reinforced by recent reports demonstrating that, in addition to classical EMT inducers, CXCR4's ligand, CXCL12, drives Twist-dependent EMT-like events in human glioblastoma cells (Yao et al., 2016), as well as EMT in numerous peripheral cancers (Hu et al., 2014; Li et al., 2014; Roccaro et al., 2015) and UT's ligand, urotensin II, promotes the expression of EMT markers in renal tubular epithelial cells (Pang et al., 2016).

## Concluding remarks

Although there are still many gaps in our understanding of how Atg proteins control chemotactic migration and cancer cell invasion, it is now clear that the autophagy machinery has major impacts on these processes. Specifically, degradation of focal adhesion components, through selective autophagy, has already been shown to participate in the turnover of adhesions during cancer cell migration. Autophagic degradation of key proteins participating in actin remodeling may also constitute an efficient way of clearing these proteins from the cell rear and concentrating them at the cell front, in order to initiate the expansion of a single lamellipodium in the direction of the chemotactic stimulus. The recent identification of the plasma membrane as a donor compartment for the expansion of the phagophore constituted an essential step in the comprehension of how chemotactic receptors could locally control autophagic flux. Deciphering the signaling cascades triggered by these receptors, and their impacts on the trafficking and/or processing of the core Atg proteins is an exciting challenge for the future and will help to envisage innovative strategies to halt cancer metastasis.

## Author contributions

PC, PG, HC, and FM wrote manuscript.

## Funding

This work was supported by INSERM, Gefluc, TC2N network, the Ligue Contre le Cancer Normandie, the French Agence Nationale de la Recherche, and the University of Rouen. PC is recipient of a fellowship from the French ministry.

### Conflict of interest statement

The authors declare that the research was conducted in the absence of any commercial or financial relationships that could be construed as a potential conflict of interest.

## Abbreviations

CXCR4: C-X-C motif chemokine receptor 4
DFC1: double FYVE domain- containing protein 1
ECM: extracellular matrix
EMT: epithelial-to-mesenchymal transition
ER: endoplasmic reticulum
ERGIC: ER-Golgi intermediate compartment
GPCR: G protein-coupled receptor
LC3: microtubule associated protein 1 light chain 3
mTOR: mechanistic target of rapamycin (serine/threonine kinase)
NBR1: neighbor of BRCA1 gene 1
PI3K: phosphatidylinositol 3-kinase
PI3P: phosphatidylinositol 3-phosphate
PIP3: phosphatidylinositol (3,4,5)-triphosphate
PKA: protein kinase A
UT: urotensin II receptor
WIPI: WD repeat domain phosphoinositide interacting protein.
